# Significance of genetic modifiers of hemoglobinopathies leading towards precision medicine

**DOI:** 10.1038/s41598-021-00169-x

**Published:** 2021-10-22

**Authors:** Priya Hariharan, Manju Gorivale, Pratibha Sawant, Pallavi Mehta, Anita Nadkarni

**Affiliations:** Department of Haematogenetics, ICMR-National Institute of Immunohaematology, 13th Floor NMS Building, KEM Hospital Campus, Parel, Mumbai, 400012 India

**Keywords:** Genetics, Molecular biology, Diseases, Molecular medicine

## Abstract

Hemoglobinopathies though a monogenic disorder, show phenotypic variability. Hence, understanding the genetics underlying the heritable sub-phenotypes of hemoglobinopathies, specific to each population, would be prognostically useful and could inform personalized therapeutics. This study aimed to evaluate the role of genetic modifiers leading to higher HbF production with cumulative impact of the modifiers on disease severity. 200 patients (100 β-thalassemia homozygotes, 100 Sickle Cell Anemia), and 50 healthy controls were recruited. Primary screening followed with molecular analysis for confirming the β-hemoglobinopathy was performed. Co-existing α-thalassemia and the polymorphisms located in 3 genetic loci linked to HbF regulation were screened. The most remarkable result was the association of SNPs with clinically relevant phenotypic groups. The γ-globin gene promoter polymorphisms [− 158 C → T, + 25 G → A],BCL11A rs1427407 G → T, − 3 bp HBS1L-MYB rs66650371 and rs9399137 T → C polymorphisms were correlated with higher HbF, in group that has lower disease severity score (P < 0.00001), milder clinical presentation, and a significant delay in the age of the first transfusion. Our study emphasizes the complex genetic interactions underlying the disease phenotype that may be a prognostic marker for predicting the clinical severity and assist in disease management.

## Introduction

β-Thalassemia and sickle cell anemia (SCA) form a major health burden in India as they cause a high degree of morbidity, moderate to severe hemolytic anemia with the carrier frequency varying from 3 to 17% among different population groups of India^1^. Both the diseases are caused by mutations in the β-globin gene and are inherited as an autosomal recessive single gene disorder. However, despite this apparent genetic simplicity, both the disorders display a remarkable spectrum of phenotypic severity. In β-thalassemics, the primary determinant of disease severity is the type of β-globin gene mutation that the patient inherits. Severe β-thalassemia mutation (β^0^/β^+^) may completely down-regulate β-globin chain synthesis. However, milder β^++^ mutations, present in the conserved 5′ β-globin promoter region and 3′ untranslated region, may alter the mRNA expression, leading to the moderate synthesis of the β-globin chain^2^. It has been observed that patients inheriting the same β-globin gene mutations, display phenotypic heterogeneity. Thus, the clinical variability observed among the patients prompted the search for the additional genetic modulators of these diseases.

Several modifier genes have been identified which influence the severity of hemoglobinopathies. The most common secondary modifier is the co-inheritance of α-thalassemia and the elevated levels of fetal hemoglobin (HbF), both of which directly reduce the globin chain imbalance. An interacting combination of α-thalassemia with hemoglobinopathy has been shown to alleviate the severity by reducing the intracellular precipitation of free alpha-globin chains in β-thalassemia and by reducing HbS concentration in SCA patients^3,4^.

The possibility that the severity of hemoglobinopathies could be reduced by induction of HbF was realized as the symptoms of hemoglobinopathies are observed after 6 months of age after the birth when there is a gradual reduction in the HbF levels. Thus, the study of other secondary mechanisms related to the sustained production of HbF in adult life may be important. With the advent of genome-wide association studies, recently several unlinked genetic factors have been identified which elevate the HbF levels^5^. Four major quantitative trait loci (QTLs): the beta globin gene (HBB) locus, the B-cell lymphoma/leukemia 11A (BCL11A)gene, the HBS1 Like Translational GTPase-MYB Proto-Oncogene (HBSB1L-cMYB) inter-region along with Krueppel-like factor 1 (KLF1) gene variations bring about 20–50% variation in HbF levels in hemoglobinopathy patients^5^. Thus, in this study, we have screened for the presence of primary and secondary modifiers in hemoglobinopathy patients and have analysed the cumulative effect of these modifiers on the phenotypic variability in the patient group.

## Materials and methods

### Selection of patients and healthy controls

The study was approved by the National Institute of Immunohaematology-Institutional Ethics Committee. All methods were performed in accordance with relevant guidelines and regulation. Unrelated 100 β-thalassemia homozygous and 100 SCA patients were selected for this study. As this study aimed to determine the correlation between genetic modifiers of the β-hemoglobinopaties with the phenotypic manifestations of the patients, among the β-thalassemia homozygous group, 50 patients with severe phenotype (β-thalassemia major) and 50 patients with milder phenotype (β-thalassemia intermedia) were specifically selected. 50 unrelated healthy adult blood donors with normal hematological indices, with no transfusion history and medication, were randomly selected. The blood samples were collected in K2 EDTA vacutainers after informed consent.

### Primary screening and molecular analysis

Primary screening involved complete blood count analysis and the concentration of different hemoglobin fractions was quantified on BioRad Variant II high-performance liquid chromatography.

Molecular analysis of the β-globin gene was first carried out to confirm the hemoglobinopathy status in the patient samples by covalent reverse dot blot hybridization (CRDB), amplification refractory mutation system—polymerase chain reaction (AMRS PCR), or by direct DNA sequencing^6^. α-globin gene deletions were detected by multiplex PCR^7^. The γ-globin promoter region [NC_000011.9 (5250415–5249612)] was screened by direct DNA sequencing. Five BCL11A polymorphisms which showed the highest correlation with HbF levels [rs11886868C → T (NC_000002.12:g.60493111C > T) [Assay ID: C__11363852_10], rs7557939 A → G] (NC_000002.12:g.60494212G > A) [Assay ID: C___2069774_10], rs4671393 A → G NC_000002.12:g.60493816A > G [Assay ID: C__25926414_10], rs1427407 G → T (NC_000002.12:g.60490908T > G) [Primers: Common F: 5′ATGTGTTCCCAATGAGTTTC3′, Reverse Normal allele: 5′GTTCAAGTAGATATCAGAAGGGAGA3′, Reverse Mutant allele: 5′GTTCAAGTAGATATCAGAAGGGAGC3′] and rs7606173 G → C (NC_000002.12:g.60498316G > C) [Primers: Forward Normal allele: 5′GCTGGGCACAGCCTTGAC3′, Forward Mutant allele: 5′GCTGGGCACAGCCTTGAG3′, Common Reverse: 5′CTAGGAAGGGAAGTGGGTAT3′] were analyzed by real-time SNP genotyping^8^ and by ARMS PCR. 2 polymorphisms in HBS1L-MYB intergenic region [rs66650371 Intact ‘TAC’ → Deletion ‘TAC’ (NC_000006.12:g.135097497_135097499del) [Primers for detecting normal allele: Forward primer: 5′TCACTCTGGACAGCAGATGTTACTAT3′, Reverse primer: 5′CTCAGTGATGGTATTTCTGGAGAC3′, Primers for detecting mutant allele: Forward primer: 5′AGCCCGTCCAGACACTCATTGTT3′, Reverse primer: 5′GCCCTGATAACATTTTGTGGTTTTCATTTAACAT3′], rs9399137 T* → *C (NC_000006.12:g.135097880T > C)] were screened by ARMS PCR and by DNA sequencing respectively.

The patients were clinically evaluated and the disease severity score was calculated based on the detailed clinical history of the patient^9,10^. Linkage disequilibrium analysis was performed by using Haploview software. (https://www.broadinstitute.org/haploview/haploview).

### Statistical analysis

Statistical analysis of the data was performed using GraphPad version 6.01 software (Graph Pad Prism Inc, California, U.S.A). The hematological indices among different patient groups and normal controls are represented as mean ± standard deviation (SD). Fischer extract test was used to compare the polymorphism distribution among the patients and the control groups. The comparison of the quantitative variables among the groups and between differing genotypes was carried out by unpaired non-parametric Mann–Whitney U test. The P-value ≤ 0.05 was considered to be statistically significant. Generalized Multifactor Dimensionality Reduction (GMDR) software version beta 0.9 was used to analyse the interaction among the SNPs in different patient groups. The Kaplan Meier survival curve analysis was performed to determine the age of presentation by considering the transfusion free survival among the patient groups.

### Ethics approval

The study was approved by the National Institute of Immunohaematology-Institutional Ethics Committee.

### Consent to participate

Informed consent was obtained from all individual participants included in the study.

## Results

On the basis of clinical history, the β-thalassemia patients were classified into a severe group (50 Thalassemia major: TM) and milder group (50 Thalassemia Intermedia: TI).

3 parameters were considered for clinical analysis in both the hemoglobinopathy patient group. These included the age of presentation, frequency of blood transfusion, and organomegaly. As expected, the patients in the β-thalassemia major group had an early age of presentation (9.2 ± 2.7 months) and recurrent blood transfusion requirement (14.8 ± 4.0 times/year) as compared to the milder β-thalassemia intermedia group (mean age: 4.5 ± 3.3 years and transfusion frequency: 2.5 ± 3.3 times/year). In the SCA, the mean age of presentation was found to be 6.3 ± 5.2 years. Further to know the contribution of HbF levels in the clinical presentation of the SCA patients, they were divided into two groups considering the median HbF level of 17.4%. It was observed that SCA patients with HbF levels ≤ 17.4% showed higher mean transfusion frequency (3.3 ± 5.5 times/year) as compared to patients with HbF > 17.4% (1.7 ± 5.4 times/year). Hepatosplenomegaly was pronounced in β-thalassemia intermedia group as against thalassemia major (P: 0.001). Also, in SCA patients, hepatosplenomegaly was observed. The detailed clinical analysis among the patient groups is shown in Table 1. The hematological parameters of the patient groups are shown in Table 2.

**Table 1:** Clinical characteristics among the patient groups.

Parameters	β-thalassemia Homozygous		Sickle cell anemia patients	
	β-thalassemia major n = 50	β -thalassemia intermedia n = 50	HbF level: ≤ 17.4% n = 50	HbF level : > 17.4% n = 50
**Mean ± SD**				
Age of presentation	9.2 ± 2.7 months	4.5 ± 3.3 years	5.2 ± 4.9	7.1 ± 5.5
Blood transfusion per year (no. of times)	14.8 ± 4.0	2.5 ± 3.3	3.3 ± 5.5	1.7 ± 5.4
Hepatomegaly (cm)^a^	2.7 ± 2.9	4.6 ± 3.5	5.3 ± 3.1	
Splenomegaly (cm)^b^	4.7 ± 1.7	6.5 ± 3.6	6.8 ± 5.1	

*n* Number of patients.

^a^Hepatomegaly pronounced in thalassemia intermedia and Sickle cell anemia patients.

^b^Splenomegaly in thalassemia intermedia and Sickle cell anemia patients.

**Table 2:** Hematological analysis among the patient and the control groups.

Parameters	Controls	β-thalassemia homozygous			Sickle cell anemia patients		
	Mean ± SD	TM, n: 27 Mean ± SD ^a^	TI, n: 50 Mean ± SD	*P value 95% CI	HbF ≤ 17.4% n: 50 Mean ± SD	HbF > 17.4% n: 50 Mean ± SD	**P value 95% CI
RBCs (10^6^/µL)	4.6 ± 0.6	2.8 ± 0.70	3.5 ± 0.69	< 0.0001	3.1 ± 0.9	3.1 ± 0.9	0.5
Hb (g/dL)	13.5 ± 2.04	5.4 ± 1.17	7.8 ± 1.4	< 0.0001	8.1 ± 2.1	9.1 ± 2.5	0.03
MCV (fL)	83.9 ± 4.2	75.1 ± 17.3	70.7 ± 7.8	0.92	79.4 ± 12.0	84.0 ± 10.6	0.04
MCH (pg)	28.9 ± 4.2	24.4 ± 5.2	22.8 ± 3.4	0.28	26.4 ± 4.4	29.2 ± 3.8	0.008
MCHC (g/dL)	34.5 ± 1.5	31.6 ± 3.5	32.2 ± 1.9	0.18	33.2 ± 2.1	34.8 ± 2.5	0.0003
RDW (%)	13.85 ± 1.5	32.8 ± 9.8	30.8 ± 5.9	0.36	22.8 ± 4.6	20.8 ± 6.3	0.0016
Platelets (10^3^/µL)	291.7 ± 38.0	333 ± 246.8	370.8 ± 269	0.23	349.7 ± 211.1	221 ± 115.6	0.0002
HbA_2_ (%)	2.6 ± 0.2	3.6 ± 1.5	3.4 ± 1.9	0.08	3.0 ± 0.5	2.9 ± 1.1	0.007
HbF (%)	0.31 ± 0.2	65.6 ± 36.3	66.3 ± 34.2	0.46	12.7 ± 3.7	26.4 ± 7.1	< 0.0001
F-cells (%)	2.4 ± 0.5	67.1 ± 26.01	68.0 ± 24.1	0.81	31.8 ± 18.6	43.2 ± 18.2	0.06
HbS (%)	–	–	–	–	74.1 ± 11.7	67.5 ± 9.4	< 0.0001

*P value calculated among the β-thalassemia homozygous groups, **P value calculated among the SCA patient groups. The P value was calculated using unpaired non parametric Mann–Whitney U test.

^a^At the time of testing 23 TM patients were on regular transfusions, hence these patients were excluded from hematological analysis.

Molecular analysis of the β-globin gene showed that IVS 1–5 G → C (HBB:c.92 + 5G > C) was the commonest mutation encountered in both the β-thalassemia homozygous group (TM: 32%, TI: 28%). The six common Indian mutations [IVS 1–5 (G → C), 619 bp deletion (NG_000007.3:g.71609_72227del619), IVS 1–1 G → T (HBB:c.92 + 1G > T), codons 8/9 + G (HBB:c.27_28insG), codon 15 G → A (HBB:c.47G > A), codons 41/42-CTTT (HBB:c.126_129delCTTT)] accounted for 82.5% of molecular lesions (TM: 44.5%, TI: 38%). It was observed that the overall prevalence of milder mutations was higher in TI patients (8.0%) as compared to TM patients (1.0%, P: 0.07) (Supplementary Table 1).

As co-inheritance of α-thalassemia is a well-known disease modifier of β-thalassemia and SCA, the presence of α-globin-gene deletions was screened in the patient groups. A much higher prevalence of single alpha globin gene deletions was observed in SCA patients (51.0%). Among the β-thalassemia homozygotes, the β-thalassemia intermedia showed a higher prevalence (26.0%) of α-globin gene deletions as compared to β-thalassemia major (20.0%). (P: 0.47) (Supplementary Table 2).

The second powerful modifier of disease severity in hemoglobinopathy patients is elevated HbF levels. Hence the polymorphisms located in the three loci linked to raised HbF levels: γ-globin promoter region, BCL11A and HBSL1-MYB intergenic region were analysed in this study.

**Figure 1:**
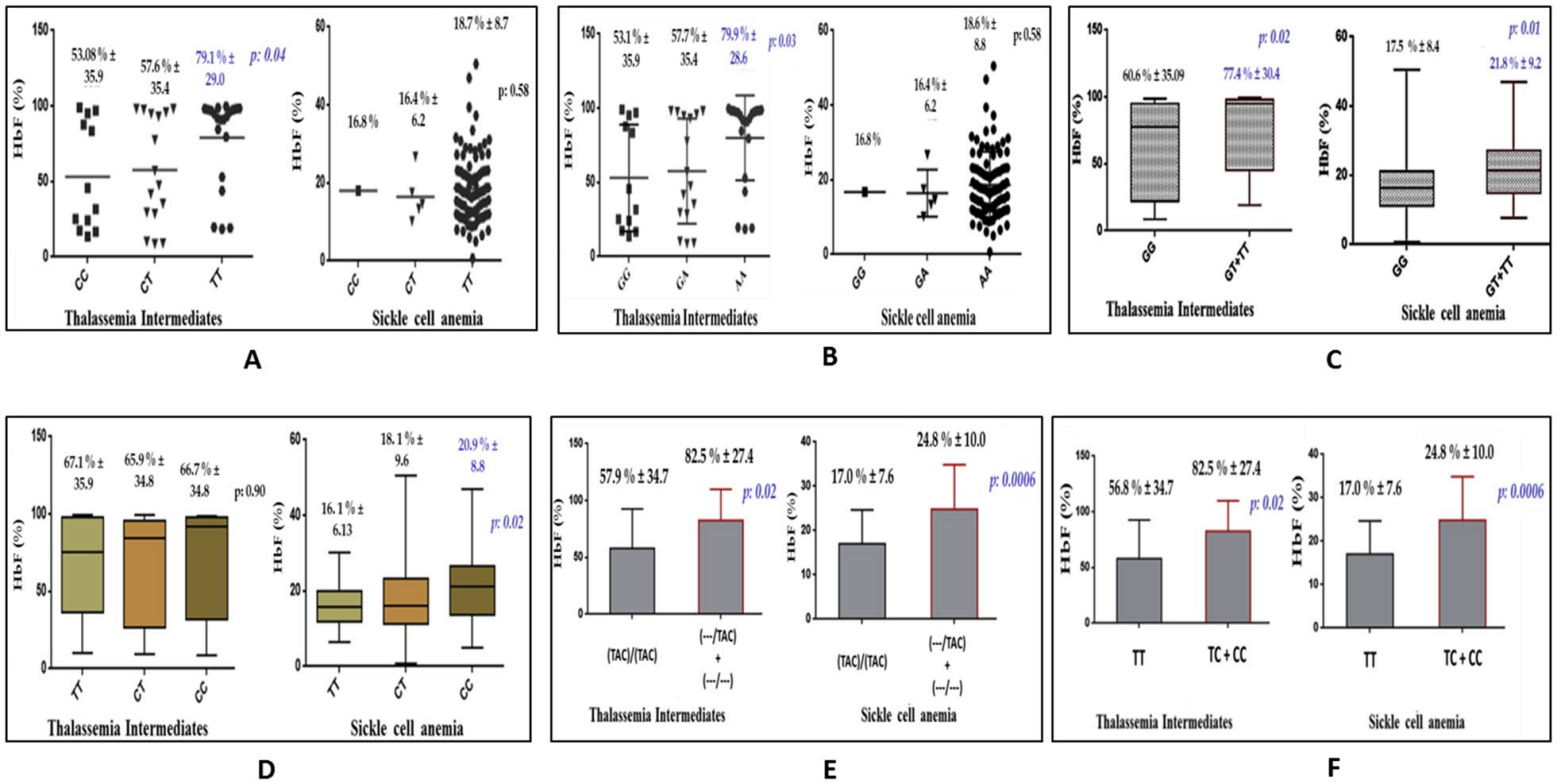
Association of the SNP genotypes with HbF levels in β-hemoglobinopathy patients. (**A**) XmnI polymorphism: − 158 (C → T), (**B**) + 25 (G → A) polymorphism, (**C**) rs1427407 (G → T) polymorphism, (**D**) rs11886868 (C → T), (**E**) rs66650371 (Intact TAC → 3 bp deletion) polymorphism, (**F**) rs9399137 (T → C) polymorphism.

In the ^G^γ globin promoter region, the XmnI polymorphism residing in the − 158 C → T (HBG2 c.-211 C → T) position was only detected. In the β-thalassemia homozygous group, the homozygosity for the mutant T allele [T/T, Xmn I+/+] was significantly higher in TI (44.0%) as compared to TM (28.0%), (P: 0.01).Similarly, in SCA patients, 94% of the patients were homozygous for the T allele. As thalassemia major patients were on recurrent transfusion, the genotypes could not be compared with the HbF levels, however, in thalassemia intermediates, the TT [XmnI: +/+] genotype was found to be significantly associated with raised HbF levels (79.1% ± 29.0, P: 0.04) as compared to the CC [XmnI: −/−] genotype (53.08% ± 35.9). (Fig. 1A).

In the ^A^γ-globin promoter region, + 25 (G → A) (HBG1:c.-29 G > A) variation was detected and A allele was found to be the variant allele. The A allele was found to be significantly higher in the TI group as compared to TM (P: 0.005). Also, the A allele in β-thalassemia intermediates, was significantly associated with increased HbF levels (79.9% ± 28.6, P: 0.03). In SCA patients, 94% of the patients were homozygous for the A allele, an observation similar to the XmnI polymorphism (Fig. 1B).

Among the 5 intronic polymorphisms in the BCL11A gene screened, the mutant T allele of rs1427407 (G → T) polymorphism, was significantly higher in the β-thalassemia intermedia group as compared to the thalassemia major group (P: 0.002, OR 5.6, 1.84–17.22). In SCA patients the T allele was found to be significantly associated with raised HbF levels (HBF > 17.4% (P: 0.003, OR 3.14, 1.46–6.75) as compared to the other group. The T allele was also found to be significantly associated with HbF levels in both the patient groups (P < 0.05) (Fig. 1C).

In the sickle cell anemia patients, the C allele of rs11886868 C → T polymorphism was found to be significantly associated with increased HbF levels (P: 0.02, HbF: 20.9% ± 8.8) (Fig. 1D). Among the HBS1L-MYB intergenic polymorphisms, the deletional allele of rs66650371 (Intact TAC → Deletion—‘TAC’) polymorphism and the C allele of rs9399137 (T → C) were found to be significantly present in the milder β-hemoglobinopathy patients. As reported in earlier studies these 2 polymorphisms were found to be in complete linkage disequilibrium. The minor alleles of these polymorphisms were found to be significantly associated with the HbF levels (Fig. 1E,F). Table 3 gives a detailed analysis of the allelic frequency of these polymorphisms determined among the patient and the control group.

**Table 3:** Genotypic and allelic frequency of the polymorphisms in patient and control group.

Allelic frequencies (N: 100)		Controls n:50	β-Thalassemia homozygous		Sickle cell anemia patients		*P value/Odds Ratio 95% CI	**P value/Odds Ratio 95% CI
			TM n:50	TI n:50	HbF ≤ 17.4% n:50	HbF > 17.4% n:50		
− 158 (C → T)	C	81	59	41	5	2	0.01/2.07 (1.1–3.6)	0.29/2.4 (0.4–12.7)
	T	19	41	59	95	98		
+ 25 (G → A)	G	81	60	40	5	2	0.005/2.2 (1.27–3.96)	0.29/2.4 (0.4–12.7)
	A	19	40	60	95	98		
rs11886868 (C → T)	C	60	55	59	47	57	0.56/1.17 (0.67–2.06)	0.11/1.56 (0.89–2.7)
	T	40	45	41	53	43		
rs7557939 (A → G)	A	38	42	43	47	36	0.88/1.04 (0.5–1.8)	0.11/1.57 (0.9–2.7)
	G	62	58	57	53	64		
rs4671393 (A → G)	A	26	12	18	30	31	0.2/1.6 (0.73–3.5)	0.95/1.01 (0.55–1.85)
	G	74	88	82	70	69		
rs1427407 (G → T)	G	95	96	81	89	72	0.002/5.6 (1.84–17.2)	0.003/3.14 (1.46–6.75)
	**T**	5	4	19	11	28		
rs7606173 (G → C)	G	93	93	95	80	78	0.55/0.69 (0.21–2.28)	0.72/1.1 (0.57–2.22)
	C	07	7	5	20	22		
rs66650371 TAC → 3 base Deletion	TAC	85	86	75	94	79	0.06/2.0 (0.96–4.12)	0.007/3.76 (1.4–9.8)
	**–**	15	14	25	6	21		
rs9399137 (T → C)	T	85	86	75	94	79	0.06/2.0 (0.96–4.12)	0.007/3.76 (1.4–9.8)
	C	15	14	25	6	21		

*n* Number of patients in each group, *N* Total number of alleles in each group.

*Thalassemia patients.

**SCA patients.

**Figure 2:**
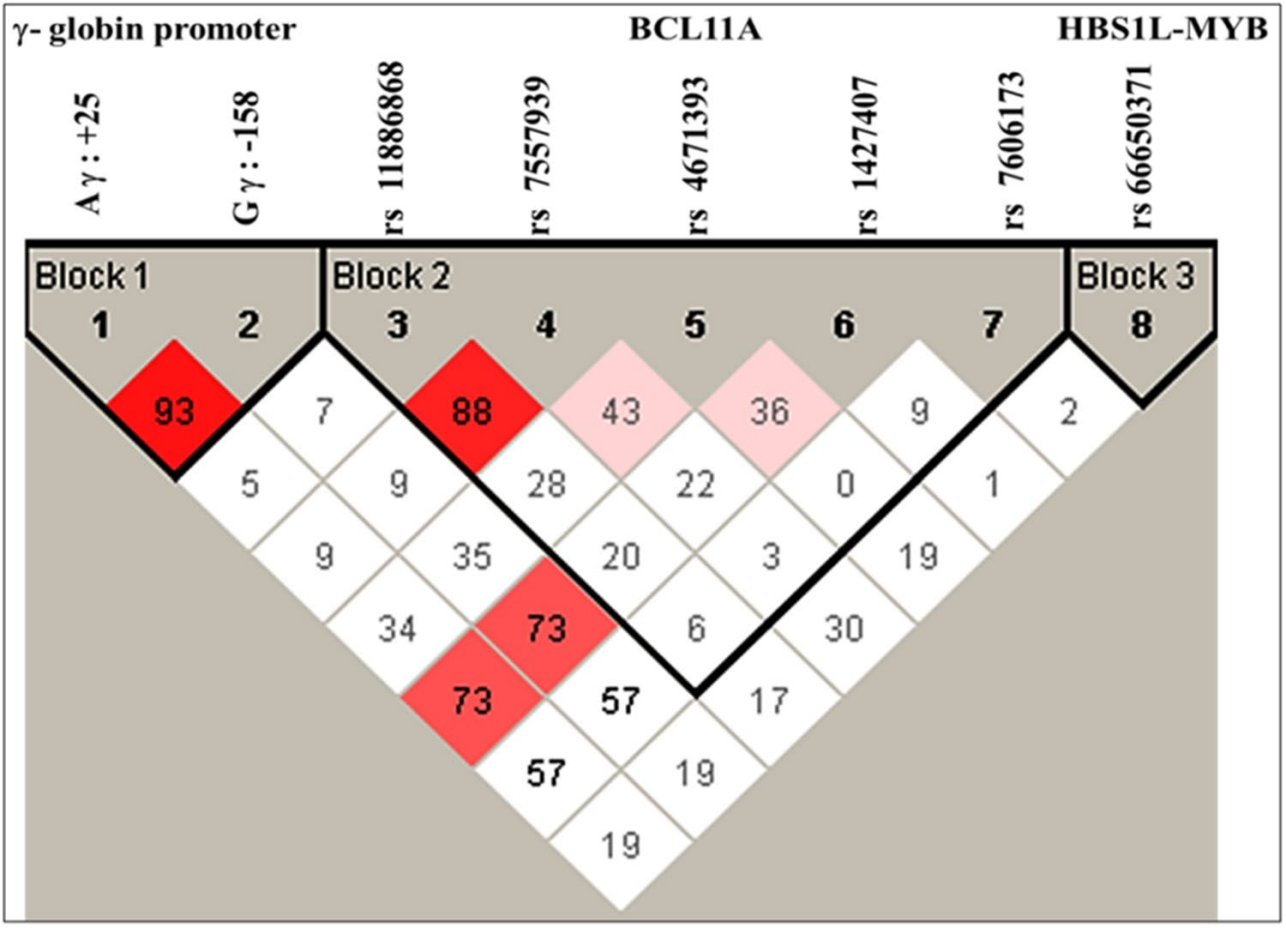
Linkage disequilibrium (LD) plot of the SNPs. It could be observed that the + 25 (G → A) polymorphism in ^A^γ-globin gene and the XmnI polymorphism (− 158 C → T) in the ^G^γ-globin are highly linked (D′: 93). Also the SNPs rs11886868 (C → T) and rs7557939 (A → G) in the BCL11A intronic region, are strongly linked (D′: 88) with each other.

The linkage disequilibrium plot showed that the + 25 (G → A) polymorphism in ^A^γ-globin gene and the XmnI polymorphism in the ^G^γ-globin are highly linked (Linkage Disequilibrium coefficient D′: 93). Also, the SNPs rs11886868 (C → T) and rs7557939 (A → G) in the BCL11A intronic region, are strongly linked (D′: 88) with each other (Fig. 2).

**Figure 3:**
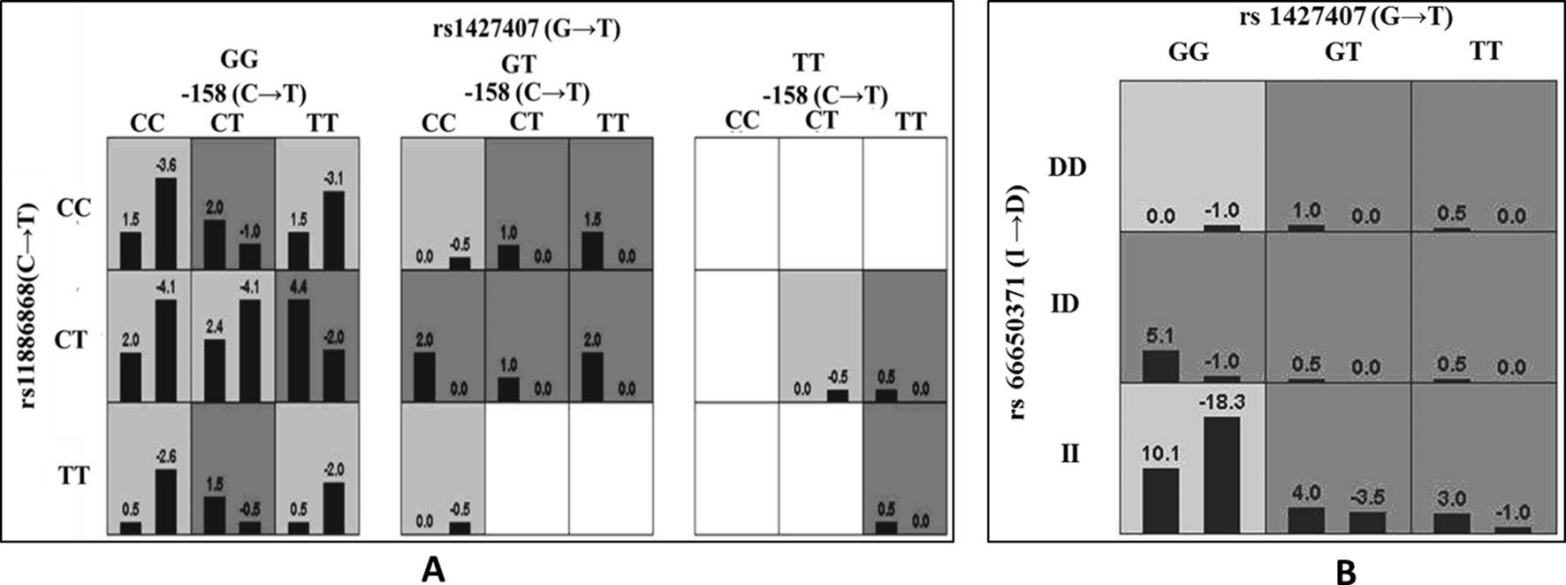
SNP tile analysis of gene–gene interaction in β-hemoglobinopathy patients. The tiles are shaded according to the cumulative scores of both the bars. Here the dark grey tiles represent a positive score (presence of mutant HbF boosting allele in milder patient group) and the light grey tiles represent a negative score (presence of normal allele in the severe patient group). (**A**) The mutant alleles of 3 SNPs (XmnI polymorphism, rs11886868(C → T) and rs1427407 (G → T) found to be significantly higher in β-thalassemia intermedia patients (the left bar) as compared to the β-thalassemia major patients (right bar). (**B**) The mutant alleles 2 SNPs [rs66650371 [TAC (I) > deletion of 3 bp (D)] and rs1427407 (G → T)] found to be significantly higher in SCA patients with HbF levels > 17.4% (left bar) as compared to the SCA patients with HbF ≤ 17.4% (right bar).

Further, the best SNP models accompanied by the lowest prediction error (Testing balance accuracy), the highest CVC, and the P-value of significant level were calculated. The results revealed a cumulative effect of the mutant alleles of the 3 SNPs − 158(C → T), rs11886868 (C → T), and rs1427407 (G → T) significantly higher in β-thalassemia intermedia patients, and as the best SNP model with testing balance accuracy of 74.9% and cross-validation consistency of 9/10. Further gene–gene interaction studies showed a synergistic effect may coexist among these SNPs in elevating the HbF level (Fig. 3A). Similarly among the SCA patients, gene–gene interaction between the mutant alleles of rs66650371 and rs1427407 were found to be significantly higher in the sickle cell anemia patients with HbF levels > 17.4% with a testing balance accuracy 66.0% and cross-validation consistency 10/10 (Fig. 3B). The generation of GMDR models for determining the most influential SNPs among the 9 SNPs studied in the patient groups is shown in Supplementary Table 3.

**Figure 4:**
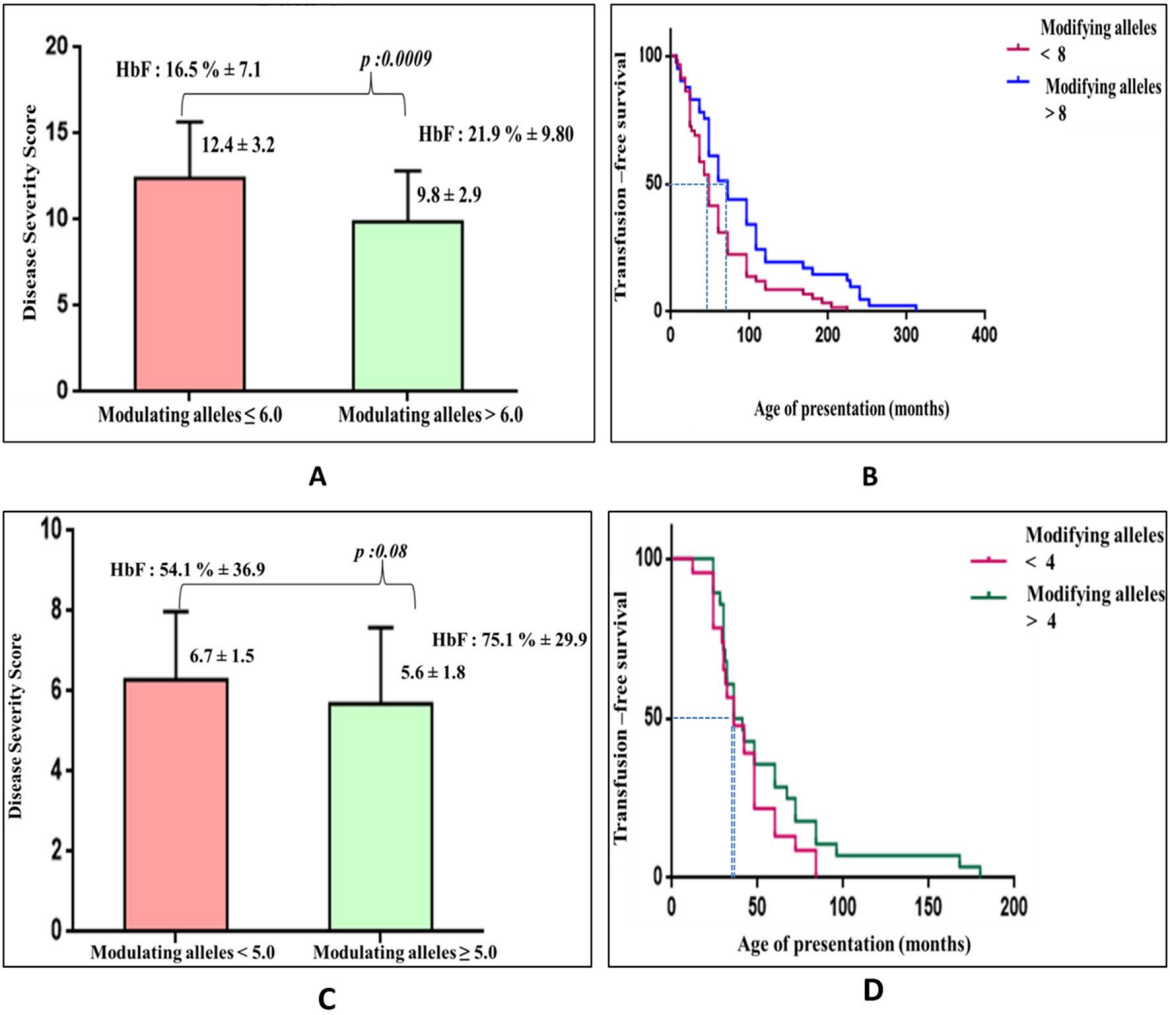
Association of disease ameliorating alleles with disease severity in the patients. (**A**) Depicts the disease severity score against the median number of HbF modulating alleles in the SCA patients. The patients with higher number of modulating alleles showed significant positive correlation with the HbF levels (mean HbF: 21.9% ± 9.8) as compared to the other patient group. (**B**) Kaplan Meier transfusion free survival curve was plotted considering all the genetic modifiers (α-thalassemia and the HbF modifiers) and a delayed median age of presentation in patients with more number of modifying alleles: 72 months was observed as compared to the other group: 48 months (P: 0.02, Gehan Breslow Wilcoxon test). (**C**) Depicts the disease severity score (DSS) against the number of HbF modulating alleles in the β-thalassemia intermedia patients. The patients inheriting more number of the disease ameliorating alleles showed elevated HbF levels (mean HbF: 75.1% ± 29.9), with reduced disease severity score (mean DSS: 5.6) as compared to the patients in the other group (P: 0.08). (**D**) Kaplan–Meier transfusion free survival curve analysis (considering both primary and secondary modifiers) showing slight delay in median age of presentation in patients with more number of modifying alleles (38.5 months) as compared to group (36.0 months) (P: 0.36, Gehan Breslow Wilcoxon test).

The presence of ameliorating alleles may significantly delay the age of presentation and transfusion requirement in β-hemoglobinopathy patients. Hence, for the analysis we included both primary modifiers (the type of β-globin gene mutation in β-thalassemia patients) and secondary modifiers: α-globin genotype and the HbF modulators [γ-globin promoter variations, BCL11A, MYB and KLF1 variations (the KLF1 data from our previous published paper)^11^. Among the SCA patients, a strong negative correlation was observed between the HbF levels and the disease severity score. (Pearson correlation coefficient r: − 0.7, P < 0.00001) The patients inheriting the higher numbers of modulating allele showed significantly elevated HbF levels (mean HbF: 21.9% ± 9.8) as compared to patients with less number of disease severity modulating alleles (mean HbF: 16.5% ± 7.1). Also showed a significant delay in the age of first transfusion as compared to the other group (Fig. 4A,B). The β-thalassemia intermedia patients inheriting more number of the disease ameliorating alleles showed elevated HbF levels (mean HbF: 75.1% ± 29.9), with reduced disease severity score (mean DSS: 5.6) as compared to patients with lower numbers of disease severity modulating alleles, who had lower HbF levels (mean HbF: 54.1% ± 36.9). Further, when compared to the age of first transfusion, however, no significant difference was observed among the 2 groups (Fig. 4C,D). The Supplementary Table 4 shows the median transfusion free survival and hazard ratio in both the patient groups. The number of modulating alleles, transfusion free survival ratio was inversely associated with the hazard ratio.

## Discussion

Though β-thalassemia and sickle cell disease are single-gene disorders with prototypical Mendalian inheritance patterns, both the disorders display a wide spectrum of clinical phenotypes. Thus, the search for the genetic modifiers was triggered, as 5–10% of β-thalassemia homozygous patients with the same β-globin gene mutation and sickle cell anemia patients showed a variable pattern of clinical expression^12^.

In this study, we first classified the β -thalassemia patients according to the clinical severity and then studied the influence of the genetic modifiers. Modell and Berdukas^13^, reported that 60% of β-thalassemia homozygous patients presented in the first year of life, these patients were segregated as β-thalassemia major and 9% of the β-thalassemia homozygous patients who presented after 2 years of age, with intermediate clinical severity were classified as β-thalassemia intermedia^13,14^. A similar observation was made in our study, in which the β-thalassemia major patients presented early by 9.2 ± 2.7 months and the patients in the β-thalassemia intermedia group had a delayed age of presentation mean of 4.3 ± 3.3 years. The β-thalassemia intermedia patients also showed a significantly higher mean baseline hemoglobin of 7.8 ± 1.4 g/dL as compared to thalassemia major patients. Similarly, another study showed that in 63 β-thalassemia intermedia patients, the hemoglobin values ranged between 7 and 9 g/dL with occasional transfusion regimen and splenomegaly^15^. In our study as well, pronounced hepatosplenomegaly was observed in β-thalassemia intermedia patients as compared to β-thalassemia major. Mpalampa et al.^16^ considering the mean HbF cut-off as 10%, in 216 sickle cell anemia patients observed a strong negative correlation of HbF levels with the total number of transfusions (r = − 0.181, P: 0.004), hospitalisations rate (r = − 0.173, P: 0.006), and significant positive correlation with the age at diagnosis (r = 0.151, P: 0.013)^16^. In the Indian context, Nayak et al.^17^ studied 60 sickle cell anemia patients and observed fewer episodes of painful crises in children with high baseline HbF level as compared to children with low HbF level^17^. Correspondingly in our study as well, the mean age of diagnosis among 100 SCA patients was found to be 6.3 ± 5.2 years which is very much delayed as compared to the patient cohort studied by Mpalampa et al.^16^. This observation could be due to inherently elevated HbF levels in Indian patients mainly due to Arab-Indian haplotype which is a major determinant of HbF levels in Indian SCA patients^18^. Further, it was observed that the patients with higher HbF level had a delayed age of presentation (7.1 ± 5.5 years) with less transfusion requirement and sporadic painful crisis compared to patients with HbF level ≤ 17.4% (age of presentation: 5.2 ± 4.9 years).

As the β-thalassemia alleles inherited by the patient act as a primary modulator of the disease severity in β-thalassemia, Colah et al.^19^ observed that the milder mutations are prevalent in β-thalassemia intermedia group as compared to severe β-thalassemia major patients^19^. Similarly in our study, the presence of milder β-thalassemia alleles were significantly higher in β-thalassemia intermedia as compared to β-thalassemia major patients [P: 0.004, Odds Ratio 8.6 (95% CI 1.9–37.9)]. However, Garewal et al.^20^ described that, in majority of the Indian patients, the beta genotype alone cannot predict the clinical phenotype of the patients^20^. A similar observation was seen in our patients, which suggested the presence of other genetic factors that may play a synergistic role in modifying the disease severity of β-thalassemia. In a previous study by Nadkarni et al.^21^, the associated α-thalassemia was found to be significantly higher in the thalassemia intermedia group (37%) as compared to β-thalassemia major group (5%) (P < 0.025)^21^. A study by Pandey et al.^22^ revealed 32% sickle cell anemia patients with co-existing α-globin gene deletion, showed a relatively milder clinical course with improved hematological indices and reduced transfusion history^22^. Similarly, Rumaney et al.^23^ observed that in Cameroon sickle cell disease patients, co-inheritance of α-thalassemia showed improved hematological indices with a better survival rate^23^. Similarly in this study, we observed that the coinheritance of α-thalassemia was higher in the milder β-thalassemia patient group as compared to the other group. 51% of SCA patients also showed presence of α-thalassemia. Alternatively, the excess alpha-globin chains play a significant role in the pathophysiology of homozygous beta-thalassaemia. The coexistence of a triplicated α-globin gene is found to be exacerbating the phenotypic severity of β-thalassemia by causing more globin chain imbalance, thus causing severe anemia^24^.

The effect of the genetic modifiers of fetal hemoglobin was also analysed in this study. A study in the Egyptian β-thalassemia patients showed that 83.3% of β-thalassemia intermedia cases were heterozygous for XmnI polymorphism as compared to β-thalassemia major (57.6%) and that β-thalassemia intermedia with single T allele of XmnI showed delayed age of diagnosis, raised HbF levels and milder disease phenotype as compared patients negative for the XmnI polymorphism^25^. In another study, it was also determined that the patients with homozygosity for the mutant T allele of XmnI polymorphism significantly showed higher mean HbF levels (85.5 ± 6.8%) as compared to the thalassemia intermedia patients homozygous for XmnI CC genotype (19.5% ± 29.3)^26^. A similar result was observed in our patient group where in the β-thalassemia intermedia patients homozygous for variant allele T showed significantly higher HbF level.

+ 25 G → A polymorphism in ^A^γ-globin promoter region was found to be significantly associated with elevated HbF levels in the β-thalassemia intermedia group. This polymorphism was first reported by Bianchi et al.^27^ and a strong linkage of this polymorphism with the − 158 C → T (XmnI polymorphism) was observed in their study as well^27^. It has been reported that + 25 G → A polymorphism reduces the binding efficacy of LYAR transcription factor (repressor of γ-globin gene expression) and abolishes the binding of 2 negative epigenetic regulators [DNA methyltransferase 3 alpha (DNMT3A) and protein arginine methyltransferase 5 (PRMT5)] to this promoter region^27,28^. Thus, it could be speculated that there could be a cumulative effect of mutant alleles of both XmnI polymorphism (T allele) and + 25 G → A polymorphism (A allele) in synergistically elevating the HbF levels.

The association of BCL11A polymorphisms with elevated HbF levels and their effect on amelioration of the disease phenotype was studied by Uda et al.^29^ in Sardinian β-thalassemia homozygous patients^29^. They showed that the mutant C allele of rs11886868 (C → T) formed the major allele in Sardinian population and was significantly associated with elevated HbF levels in β-thalassemia intermedia patient group. Similarly, in Indian patients Dadheech et al.^30^, determined that the C allele was significantly associated with the raised HbF levels and delayed the age of presentation in both thalassemia homozygous and SCA groups^30^. In our study, the mutant CC genotype was found to be significantly associated with HbF levels only in the sickle cell anemia patients.

Similarly, in Indonesian HbE-β-thalassemia patients inheriting variant alleles of rs11886868, rs766432 in the *BCL11A* gene, showed higher HbF levels and reduced disease severity as compared to patients with wild type alleles^31^. The second SNP that was found to be significantly associated with the HbF levels is rs1427407 (G → T) polymorphism in the *BCL11A* gene. Our results were found to be consistent with the earlier report by Bhanushali et al.^32^, who showed a similar distribution of allelic frequency of rs1427407 in Indian SCA patients^32^. Studies have demonstrated that the patients with the mutant T allele of rs1427407 (G → T) showed significantly higher HbF level, the results of which are concordant with our study, thus suggesting a crucial role of this SNP in modulating the HbF levels^32,33^.

Similarly, Chaouch et al.^34^ observed that co inheritance of the mutant C allele of the rs11886868 and the mutant A allele of the rs46713939 ameliorated the clinical phenotype of SCA patients^34^. In our study, though the A allele of rs46713939 was found to be higher in the TI group, no significant difference in the allelic frequencies among the milder and severe groups could be observed. Studies have identified a restricted 14 kb region in BCL11A intron 2 to be associated with H3-acetylation, RNA pol II activity as well as a strong GATA-I, TAL-1 binding site, all of which indicated the presence of a regulatory sequence in this region^35,36^. Thus, suggesting that the presence of a polymorphism could potentially alter the recruitment of transcription factors to this region**.**

Similarly, in our population, a 100% linkage was observed between rs66650371 and rs9399137 polymorphism. A similar observation was seen in the Tanzanian SCD patients where, both these polymorphisms in HMIP 2A block were strongly associated with HbF levels and showed a strong linkage^37^. Similarly, Lai et al.^38^ in β-thalassemia intermedia patients showed that the mutant alleles of rs9376090 (NC_000006.12:g.135090090T > C), rs7776054 (NC_000006.12:g.135097778A > G), rs9399137, rs9389268 (NC_000006.12:g.135098493A > G), rs9402685 (NC_000006.12:g.135098550T > C) in the HbS1L-MYB intergenic region and rs189984760 in the BCL11A locus, showed significant association with high HbF level^38^. Bioinformatic characterization of the 3 bp deletion polymorphism showed that this region acts as a binding site for 4 transcription factors TAL1/SCL, E47, GATA-2 and RUNX1/ AML1 all of which are important for erythroid differentiation, erythropoiesis and the presence of the mutant allele may disrupt the MYB gene expression, which is a negative regulator of the γ-globin gene expression^39,40^.

In our study, it was observed that in SCA patients, the prevalence of the T allele of rs1427407 (G → T) and the 3 bp deletional allele of rs66650371 both were significantly higher in the SCA patient group possessing higher HbF levels (HbF > 17.4%) as compared to the SCA patients, with lower HbF level (HbF ≤ 17.4%). A similar result was shown by Adeyemo et al.^41^, where in patients with the mutant alleles of these 2 polymorphisms had a milder form of the disease, with improved hemoglobin levels^41^.

Similarly, in β-thalassemia homozygous patients, the cumulative effect of 3 HbF associated mutant alleles of the SNPs − 158(C → T), rs11886868 (C → T) and rs1427407 (G → T), were observed significantly in the β-thalassemia intermedia group as compared to β-thalassemia major group. A comparable result was reported by Allawi et al.^42^, where they determined the main factors leading to milder phenotypes were the attenuated β-thalassemia alleles, the T allele of XmnI polymorphism and the minor allele of BCL11A rs10189857^42^. Cardoso et al.^43^, studied the influence of three known major loci on the HbF trait (HBG2, rs748214; BCL11A, rs4671393; and HBS1L-MYB, rs28384513, rs489544 and rs9399137) in north Brazilian SCA patients and they showed that the raised HbF trait was primarily influenced by mutant alleles of BCL11A^43^.

Further to predict the disease severity in presence of these genetic modifiers, Badens et al.^5^ studied 5 genetic modifiers of β-thalassemia. By regression analysis, all 5 types of favorable allele were found to be significantly associated with thalassemia intermedia phenotype. The β-globin gene mutations and XmnI polymorphism were the most influential modifiers of the disease severity^5^. A similar observation was reported by Danjou et al.^44^, wherein they further evaluated the age of the first transfusion with respect to the inheritance of HbF boosting alleles and observed that the age of transfusion was found to be delayed in presence of more number of HbF inducing alleles^44^. Similarly, in our study, it was observed that, 8% of thalassemia intermedia patients (HbF: 82.7% ± 22.6) had inherited more than 10 disease ameliorating alleles as compared to none in thalassemia major patients. Thalassemia major patients (64%) showed less number of disease modifying alleles as compared to the thalassemia intermedia patients (36%). Similarly, in SCA patients, the ameliorating alleles was found to be higher in the raised HbF group (HbF > 17.4) (31.3%) (mean HbF: 26.2% ± 8.3) as compared to patients in low HbF group (HbF ≤ 17.4%) (4%) (mean HbF: 12.1% ± 3.5).

These observations suggest that the presence of increased number of an ameliorating allele, may help in reducing the disease severity in hemoglobinopathy patients mainly by restoring the globin chain imbalance. The precise identification of the polymorphisms associated with elevated HbF levels may help in developing a molecular chip that may assist in predicting the disease severity. Validation of our results needs to be carried out in a bigger cohort.

## Conclusions

The present study expands the knowledge of the frequency of the genetic modifiers (primary and secondary modifiers) and the independent effect of individual predictor genes on HbF levels in hemoglobinopathy patients. The analysis of the cumulative effect of the HbF modulators may help in identifying the strongest response gene to the HbF level in both β-thalassemia and sickle cell anemia patients in the population. The predictions based on genetic modifiers thus can foresee the severity of β-thalassemia and SCA. This study may assist the clinicians, to predict the clinical phenotype of hemoglobinopathy patients at an early stage and thus may help in the efficient management of the disease. This may contribute towards molecular mechanisms of HbF regulation and the development of therapeutic approaches for β-hemoglobinopathies.

## Supplementary Information

Supplementary Tables.
