# Supplementary material for: Significance of genetic modifiers of hemoglobinopathies leading towards precision medicine
Source: Sci Rep. 2021 Oct 22;11:20906. doi: 10.1038/s41598-021-00169-x (PMC8536722; doi:10.1038/s41598-021-00169-x)
Supplement: Supplementary file 1 — Supplementary Tables. [file 41598_2021_169_MOESM1_ESM.docx]

| **Sr.**  **No.** | **β-globin gene mutations** | **50 β -Thalassemia Major (TM)**  **(n: β- mutant alleles)** | **50 β -Thalassemia Intermediate (TI)**  **(n: β- mutant alleles)** |
| --- | --- | --- | --- |
| 1. | Codon 8/9 ( +G) | 2 | 3 |
| 2. | Codon 15 (G→A) | 9 | 8 |
| 3. | Codon 16 ( -C) | 2 | 0 |
| 4. | Codon 30 (G→C) | 5 | 3 |
| 5. | Codon 41/42 (-CTTT) | 6 | 3 |
| **6.** | **IVS I-5 (G→C)** | **64** | **56** |
| 7. | IVS I-1 (G→T) | 3 | 6 |
| 8. | IVS I-1 (G→A) | 2 | 4 |
| 9. | 619 bp deletion | 5 | 1 |
| 10. | -87 (C→G) | 0 | 1 |
| 11. | -87 (C→T) | 0 | 2 |
| 12. | -30 (T→C) | 1 | 1 |
| **13.** | **CAP +1 (A→C)** | **1** | **6** |
| **14.** | **Poly A (-AATAA)** | 0 | **4** |
| **15.** | **Poly A (A→G)** | 0 | **1** |
| **16.** | **Poly A (T→C)** | 0 | **1** |
| **Total alleles** | | 100 | 100 |

**Supplementary Table 1: Summary of β-globin gene mutations identified in β-thalassemia homozygous cases**

**Supplementary Table 2: Distribution of α-globin gene deletions among the patients groups**

| **Hemoglobinopathy** | **αα /αα** | **-α ^3.7^/αα** | **-α^4.2^/αα** | **-α^3.7^/-α ^3.7^** | **-α^3.7^/-α^4.2^** |
| --- | --- | --- | --- | --- | --- |
| Thalassemia  major (n=50) | 40  (80%) | 8  (16%) | 1  (2%) | 1  (2%) | 0  (0%) |
| Thalassemia  Intermedia (n=50) | 37  (74%) | 9  (18%) | 1  (2%) | 3  (6%) | 0  (0%) |
| Sickle cell  anemia (n=100) | 49  (49%) | 30  (30%) | 1  (1%) | 18  (18%) | 2  (2%) |

**n: Number of patients in each groups**

| Model  ( n: 100, ratio:1.0) | Training Balance  Accuracy | Testing Balance Accuracy | Sign Test (p) | Cross validation consistency |
| --- | --- | --- | --- | --- |
| β-thalassemia homozygous | | | | |
| rs1427407 (G→T) | 0.639 | 0.591 | 7(0.17) | 9/10 |
| -158 (C→T)+ rs11886868(C→T) | 0.670 | 0.42 | 3(0.94) | 2/10 |
| -158 (C→T)+ rs11886868 (C→T)+ rs1427407(G→T) | **0.757** | **0****.749** | **10**  **(0.001)** | **9/10** |
| Sickle cell anemia | | | | |
| rs66650371 (I→D) | 0.616 | 0.544 | 3(0.94) | 6/10 |
| rs66650371 (I→D) +rs1427407 (G→T) | **0.686** | **0.660** | **8(0.05)** | **10/10** |
| rs4671393 (A→G) + rs66650371 (I→D)+ rs1427407(G→T) | 0.733 | 0.534 | 7(0.17) | 5/10 |

**Supplementary Table 3 : GMDR model for 9 SNPs, for selecting the most influential HbF boosting SNPs in both the patient groups**

**For rs66650371: I: Wild type: Intact 3 bp (TAC) , D: Mutant : Deletion of 3 bp (---)**

**Supplementary Table 4: Median survival and Hazard ratio in both the patient groups**

|  | Median transfusion free  Survival ratio  (95 % CI of ratio) | Hazard Ratio  (Mantel –Haenszel)  (95 % CI of ratio) |
| --- | --- | --- |
|  | **Sickle cell anemia patients** |  |
| *Group A*  *(Modulating alleles >8)* | **1.5**  **(1.06-2.23)** | **0.53**  **(0.34-0.82)** |
| *Group B*  *(Modulating Alleles <8)* | 0.66  (0.44-0.99) | 1.88  (1.20-2.93) |
|  | **β-thalassemia intermedia patients** |  |
| *Group A*  *(Modulating alleles >4)* | **1.06**  **(0.61-1.86)** | **0.69**  **(0.36-1.31)** |
| *Group B*  *(Modulating Alleles <4)* | 0.93  (0.53-1.63) | 1.44  (0.76-2.73) |
